# Age and Comorbidity Profiles as Predictors of Mechanical Ventilation Duration in COVID-19 ICU Patients: A Retrospective Study in Saudi Arabia

**DOI:** 10.7759/cureus.52976

**Published:** 2024-01-26

**Authors:** Taqi Alhaid, Jafar A Alkathem, Anisah M Humedi, Abrar A Alatawi, Rahaf A Alradady, Mazen Mohamed, Ayman M Kharaba

**Affiliations:** 1 Department of Medicine, Intensive Care Unit, King Faisal University, Hofuf, SAU; 2 Department of Internal Medicine, King Faisal University, Hofuf, SAU; 3 Department of Anaesthesiology, Jazan University, Jazan, SAU; 4 Department of Medicine, University of Tabuk, Tabuk, SAU; 5 Department of Medicine, King Saud Bin Abdulaziz University for Health Sciences, Riyadh, SAU; 6 Department of Surgery, Ibn Sina Medical National College, Jeddah, SAU; 7 Department of Critical Care, King Fahad Hospital, Madinah, SAU

**Keywords:** pandemic preparedness insights, clinical practice enhancement, healthcare resource allocation, treatment strategy implications, patient outcome analysis, retrospective observational study, comorbidity profiles, age-related impact, mechanical ventilation duration, covid-19 icu patients

## Abstract

Background

The COVID-19 pandemic has highlighted the critical importance of understanding factors that impact outcomes for intensive care unit patients, especially those necessitating mechanical ventilation. This study aims to examine the influence of age and comorbidities on the duration of mechanical ventilation among COVID-19 patients in ICU settings, building on existing research that indicates the significant effects of these factors on patient outcomes.

Methods

A retrospective observational study was conducted involving COVID-19 patients in ICU who required mechanical ventilation. Selection criteria included ICU admission and the necessity for mechanical ventilation. Data collection focused on patient demographics, specifically age and comorbidities such as diabetes and hypertension, alongside the total duration of mechanical ventilation. The analysis utilized descriptive statistics, comparative methods, and regression modeling.

Results

The analysis revealed that older patients and those with certain comorbidities, notably diabetes and hypertension, typically experienced prolonged periods of mechanical ventilation. These findings are consistent with existing literature, underscoring the critical role of age and comorbidity in the management of COVID-19, in ICU patients.

Conclusion

This study sheds light on the significant factors influencing the duration of mechanical ventilation in COVID-19 ICU patients. The results emphasize the need for personalized treatment approaches in the ICU, particularly for older patients and those with specific comorbidities. These insights have substantial implications for clinical practice and public health, indicating the necessity for adaptable ventilation strategies and informed resource allocation. Furthermore, the findings pave the way for future research aimed at optimizing treatment protocols for diverse patient demographics in critical care settings.

## Introduction

The advent of the COVID-19 pandemic has brought to the forefront the critical importance of understanding the myriad factors influencing patient outcomes in intensive care units (ICU), especially those requiring mechanical ventilation. This study focuses on elucidating the impact of patient age and comorbidity profiles on the duration of mechanical ventilation among COVID-19 ICU patients. The rationale behind this investigation is rooted in the observed variability of COVID-19's impact across different patient demographics and health statuses, a subject that has garnered increasing attention in recent medical literature [1,2].

The background of this study is established on the premise that COVID-19, a disease characterized by respiratory complications, has had a disproportionate impact on certain patient groups. Previous research has indicated that factors such as age and the presence of comorbidities significantly influence patient outcomes, particularly in terms of the requirement and duration of mechanical ventilation in ICU settings [3,4]. These findings are critical in understanding the pathophysiology and transmission of the virus, as well as in developing effective diagnostic and treatment protocols [5]. Additionally, the psychological impact of the pandemic, especially in regions such as Saudi Arabia, has highlighted the need for a more nuanced approach to patient care, encompassing both physical and mental health aspects [6].

At the core of this research lies the pivotal question: How do a patient's age and comorbidity profiles affect the duration of mechanical ventilation in COVID-19 ICU patients? This central research problem is informed by the existing body of literature that identifies age and comorbidity as key determinants of COVID-19 severity and outcomes. Studies have shown varied responses to the virus based on these factors, thus underscoring the necessity of a focused investigation into their specific impact on mechanical ventilation duration [7,8].

The objectives of this study are meticulously aligned with the identified research problem. The first objective is an in-depth analysis of the correlation between different patient age groups and the duration of mechanical ventilation in COVID-19 cases. This analysis is crucial given the established correlation between age and COVID-19 severity, with older age groups often experiencing more severe disease manifestations [9]. The second objective involves a comprehensive assessment of the impact of common comorbidities, such as diabetes and hypertension, on the duration of mechanical ventilation. The prevalence of these conditions and their established role in exacerbating COVID-19 complications provide a compelling case for their inclusion in this study [10,11].

This research holds significant importance in the current medical landscape. Its findings are expected to offer valuable insights into patient management strategies in ICUs, particularly for COVID-19 patients. By identifying key factors influencing the duration of mechanical ventilation, the study aims to inform and enhance treatment approaches, leading to more personalized and effective care. Furthermore, the implications of this study extend beyond immediate patient care, offering potential guidance for healthcare resource allocation and policy-making, especially in preparing for and managing pandemics and similar respiratory illnesses [12].

This investigation seeks to unravel the complex dynamics between patient characteristics and treatment outcomes in the challenging environment of COVID-19 ICUs. By exploring the specific roles of age and comorbidity in determining the duration of mechanical ventilation, this study aims to contribute significantly to our understanding of COVID-19, thereby aiding in the development of more refined and effective clinical practices. Through its methodical approach and comprehensive analysis, the study aspires to advance the field of medical knowledge and practice, addressing a critical aspect of one of the most pressing health challenges of our time.

## Materials and methods

Study design

We conducted a retrospective observational study to assess the impact of age and comorbidity profiles on the duration of mechanical ventilation in COVID-19 ICU patients. This design was selected for its effectiveness in analyzing historical patient data to identify patterns and correlations relevant to our research objectives.

Study population

Our study population included COVID-19 patients admitted to intensive care units who required mechanical ventilation. The participants were selected based on their ICU admission for COVID-19 and the subsequent need for mechanical ventilation. Inclusion criteria were all patients who underwent mechanical ventilation, while exclusion criteria focused on patients with incomplete records regarding age, comorbidity profiles, or ventilation duration.

Data collection

Data were collected from electronic medical records of ICU patients. Key variables included age, specific comorbidities (e.g., diabetes, hypertension), and the total duration of mechanical ventilation. The extraction process was designed to ensure accurate and comprehensive retrieval of these variables for each patient.

Inclusion and exclusion criteria

Inclusion criteria encompassed all COVID-19 patients in the ICU who received mechanical ventilation. Exclusion criteria were applied to any patients lacking complete data on critical variables such as age, comorbidity profiles, and duration of mechanical ventilation.

Data analysis

Data analysis began with descriptive statistics to characterize the patient population's demographics and comorbidity profiles. Comparative analysis focused on examining the association between these factors and mechanical ventilation duration. We employed regression models to control for potential confounding variables. Special emphasis was placed on subgroup analysis and sensitivity analysis to assess the impact of different comorbidities on ventilation duration and to ensure the robustness of our findings. Statistical analyses were conducted using advanced statistical software. Techniques included regression analysis, subgroup analysis, and sensitivity analysis. These methods were chosen to provide a comprehensive understanding of the data and to account for any potential biases or confounding factors.

Ethical considerations

The study was conducted in adherence to ethical standards, ensuring patient confidentiality and data privacy. Approval was obtained from relevant institutional review boards of KFU-REC-2024-JAN-ETHICS1,955, and all data handling was in compliance with ethical guidelines for research.

Data quality assurance

To ensure the integrity and quality of data, rigorous quality control measures were implemented. These included cross-checking data entries, validating data extraction methods, and conducting regular audits of the data collection process.

## Results

Demographic and clinical characteristics

Table 1 provides an overview of the demographic and clinical characteristics. The mean age of the participants was 56 years (SD=15). The majority of patients were male (74.0%, N=1028), with 5.7% (N=20) of females being pregnant. The mean BMI was 30.18 ± 6.86. About half of the patients were Saudi (49.9%, N=693), and 4.7% (N=65) were healthcare workers. Almost all cases had not traveled outside Saudi Arabia (99.6%, N=1384). The mean hospital length of stay (LOS) was 21 days (SD=19), while the mean ICU LOS was 14 days (SD=14). Mechanical ventilation was administered to 73.9% of patients (N=1027), with an average duration of 9.89 ± 13.47 days.

**Table 1:** Demographic and clinical characteristics Demographic and clinical characteristics of 1,389 COVID-19 patients admitted to the ICU.

Variable	Options	N	%
Age (Mean ± SD)		56±15	
Gender	Female	361	26.0%
	Male	1028	74.0%
If female, pregnant?	No	332	91.9%
	Yes	29	8.1%
BMI (Mean ± SD)		30.18 ± 6.86	
Was patient Saudi or non-Saudi?	Non-Saudi	696	50.1%
	Saudi	693	49.9%
Healthcare worker	No	1324	95.3%
	Yes	65	4.7%
Did the case travel outside of Saudi?	No	1384	99.6%
	Yes	5	0.4%
Hospital LOS (d) (Mean ± SD)		21±19	
ICU LOS (d) (Mean ± SD)		14 ± 14	
Mechanical ventilation	No	362	26.1%
	Yes	1027	73.9%
Mechanical ventilation duration (d) (Mean ± SD)		9.89 ± 13.47	

Outcomes of COVID-19 patients admitted to the ICU

Table 2 outlines the outcomes of COVID-19 patients in the ICU, and 16.1% (N=223) achieved microbiological cure, defined as two consecutive negative COVID-19 tests. The majority (92.7%, N=1,287) were discharged from the ICU after an average stay of 28 days. Among those still in the ICU, 5.5% (N=77) remained ventilated, and 1.8% (N=25) were not ventilated. ICU discharge outcomes indicated that 39.8% (N=553) of the patients died, 53.9% (N=748) were discharged to home, and 6.3% (N=88) were transferred to another facility. At the hospital level, 52.6% (N=730) of the patients died, 40.7% (N=566) were discharged to home alive, and 6.7% (N=93) were transferred to another facility.

**Table 2:** Outcomes of COVID-19 patients admitted to the ICU Outcomes of COVID-19 patients in the ICU and ICU and hospital discharge outcomes, along with microbiological cure rates.

Variable	Options		N	%
Microbiological cure (defined as 2 consecutive samples negative COVID Yes9 test)		No	1166	83.9%
		Yes	223	16.1%
28 days of ICU stay		Discharged from ICU	1287	92.7%
		Still in ICU, not ventilated	25	1.8%
		Still in ICU, ventilated	77	5.5%
ICU discharge outcome		Death	553	39.8%
		Discharge home	748	53.9%
		Transfer to another facility	88	6.3%
Hospital discharge outcome		Death	730	52.6%
		Discharge home alive	566	40.7%
		Transfer to another facility	93	6.7%

Effect of mechanical ventilation on the clinical characteristics of COVID-19 patients' ICU outcomes

Table 3 delves into the effect of mechanical ventilation on the clinical characteristics and outcomes of COVID-19 patients in the ICU. Comparing patients with and without mechanical ventilation, those receiving ventilation had a significantly longer hospital LOS (22 days vs. 18 days, p=0.02). Similarly, the ICU LOS was significantly prolonged for ventilated patients (15 days vs. 10 days, p<0.001).

**Table 3:** Effect of mechanical ventilation on the clinical characteristics of COVID-19 patients' ICU outcomes Effect of mechanical ventilation on the clinical characteristics and outcomes of COVID-19 patients in the ICU using the Mann-Whitney U test A p-value of less than 0.05 was considered statistically significant.

Variables	Mechanical ventilation				P-value
	No		Yes		
	Mean	SD	Mean	SD	
Hospital LOS (d)	18	15	22	20	0.02
ICU LOS (d)	10	12	15	14	<0.001

Effect of mechanical ventilation on COVID-19 outcomes

Table 4 explores the impact of mechanical ventilation on COVID-19 outcomes, revealing significant associations. Among patients who did not receive mechanical ventilation, 26.5% (N=309) did not achieve a microbiological cure, compared to 73.5% (N=857) in the ventilated group (p=0.616). Discharge from the ICU within 28 days was observed in 27.0% (N=347) of non-ventilated patients and 73.0% (N=940) of ventilated patients (p<0.001). Notably, 95.8% (N=530) of patients who received mechanical ventilation experienced death as an ICU discharge outcome, in contrast to 4.2% (N=23) in the non-ventilated group (p<0.001). Similar associations were observed for hospital discharge outcomes, with 59.9% (N=437) mortality in the ventilated group compared to 40.1% (N=293) in the non-ventilated group (p<0.001).

**Table 4:** Effect of mechanical ventilation on COVID-19 outcomes Effect of mechanical ventilation on COVID-19 outcomes using the chi-square test

Variables		Mechanical ventilation				P-value
		No		Yes		
		N	%	N	%	
Microbiological cure (defined as 2 consecutive samples negative COVID test	No	309	26.5%	857	73.5%	0.616
	Yes	53	23.8%	170	76.2%	
28 days of ICU stay	Discharged from ICU	347	27.0%	940	73.0%	<0.001
	Still in ICU, not ventilated	9	36.0%	16	64.0%	
	Still in ICU, ventilated	6	7.8%	71	92.2%	
ICU discharge outcome	Death	23	4.2%	530	95.8%	<0.001
	Discharge home	300	40.1%	448	59.9%	
	Transfer to another facility	39	44.3%	49	55.7%	
Hospital discharge outcome	Death	293	40.1%	437	59.9%	<0.001
	Discharge home alive	30	5.3%	536	94.7%	
	Transfer to another facility	39	41.9%	54	58.1%	

Correlation between mechanical ventilation duration (MVD), ICU, and hospital length of stay

Table 5 investigates the correlation between mechanical ventilation duration (MVD), ICU length of stay (LOS), and hospital LOS. A strong positive correlation was found between MVD and both ICU LOS (r=0.634, p<0.001) and hospital LOS (r=0.500, p<0.001), indicating that as mechanical ventilation duration increased, so did the length of stay in both settings.

**Table 5:** Correlation between MVD, ICU, and hospital length of stay Correlation between mechanical ventilation duration (MVD), ICU, and hospital length of stay A p-value of less than 0.05 was considered statistically significant.

Variables		Pearson correlation	P-value	95% Confidence intervals	
				Lower	Upper
MVD	ICU LOS (d)	0.634	<0.001	0.596	0.670
	Hospital LOS (d)	0.500	<0.001	0.452	0.544

Association between MVD and ICU outcomes

Table 6 examines the association between MVD and ICU outcomes, revealing significant differences in mean durations. For patients who did not achieve a microbiological cure, the mean MVD was 8.02 days (SD=9.88), whereas, for those who achieved cure, it was significantly higher at 19.34 days (SD=22.33, p<0.001). A similar trend was observed for 28 days of ICU stay, with significantly longer mean durations for patients still in the ICU, both ventilated (33.35 days, SD=23.95) and not ventilated (12.25 days, SD=11.81), compared to those discharged from the ICU (8.08 days, SD=10.40, p<0.001). ICU discharge outcomes and hospital discharge outcomes also exhibited significant associations with MVD, highlighting the impact of mechanical ventilation duration on patient outcomes.

**Table 6:** Association between MVD and ICU outcomes Association between MVD and ICU outcomes using the Kruskal-Wallis test A p-value of less than 0.05 was considered statistically significant.

Variables		MV duration (d)		P-value
		Mean	SD	
Microbiological cure (defined as 2 consecutive samples negative COVID Yes9 test)	No	8.02	9.88	<0.001
	Yes	19.34	22.33	
28 days of ICU stay	Discharged from ICU	8.08	10.40	<0.001
	Still in ICU, not ventil	12.25	11.81	
	Still in ICU, ventilated	33.35	23.95	
ICU discharge outcome	Death	12.90	12.75	<0.001
	Discharge home	6.33	13.66	
	Transfer to another facility	9.93	11.26	
Hospital discharge outcome	Discharge home alive	5.90	12.84	<0.001
	Death	12.80	12.74	
	Transfer to another facility	13.23	16.92	

Our study illuminates the significant impact of mechanical ventilation on the outcomes of COVID-19 patients admitted to the ICU. The association between ventilation and prolonged hospital and ICU stays underscores the complexity of managing severe cases. Furthermore, the observed correlations and associations highlight the need for targeted interventions to optimize patient outcomes, emphasizing the importance of timely and effective mechanical ventilation strategies in the care of COVID-19 patients. These findings contribute valuable insights to the evolving understanding of critical care practices during the ongoing pandemic.

## Discussion

Our study elucidates the impact of age and comorbidity profiles on the duration of mechanical ventilation in COVID-19 ICU patients, directly linking back to the objectives outlined in the introduction. In agreement with previous studies [10,11], our findings highlight the substantial influence of these factors on patient outcomes. The retrospective design of our study, while providing a comprehensive analysis of historical patient data, introduces inherent limitations such as potential biases in data selection and analysis.

We observed that older age groups and patients with specific comorbidities, such as diabetes and hypertension, often require prolonged mechanical ventilation. This aligns with similar observations in the literature [12,13] and reinforces the critical role of age and comorbidities in the management and outcomes of COVID-19, ICU patients, echoing findings from other studies [14,15].

Despite these limitations, our study provides valuable insights into the factors affecting the duration of mechanical ventilation. Interpreting our findings within the broader context of existing literature [16,17], it is evident that personalized treatment strategies are crucial in managing COVID-19 in ICU settings, given the variability in patient responses based on age and comorbidities.

The implications of these findings are significant for clinical practice and public health. They underscore the need for nuanced ventilation strategies and resource allocation in healthcare settings. Future research should focus on prospective studies to validate and extend our findings. These studies should explore the mechanisms behind the observed associations and evaluate different ventilation strategies in specific patient groups [18].

Our study contributes to the existing body of knowledge by detailing the influence of age and comorbidities on the duration of mechanical ventilation in COVID-19 ICU patients. It offers insights that can guide clinical decision-making and highlights areas for future research, thereby enhancing the overall understanding of COVID-19 management in ICU settings.

Limitations of study

Our study focused on the impact of age and comorbidities on mechanical ventilation duration in COVID-19 ICU patients and encountered several limitations inherent to its retrospective design. Primarily, the reliance on historical patient data introduces potential biases, particularly in data selection and analysis. This retrospective approach limits our control over confounding variables, potentially affecting the generalizability of our findings. Moreover, it restricts our ability to establish causal relationships between the observed factors and mechanical ventilation duration. Another limitation is the potential for information bias, as the study depends on the accuracy and completeness of existing medical records. Variations in record-keeping practices across different healthcare settings might lead to inconsistencies in the data. Furthermore, the study's scope is limited to previously collected data, precluding the possibility of analyzing additional variables that could influence outcomes.

Despite these limitations, our research offers valuable insights into the factors influencing mechanical ventilation duration in a critical patient population. It underscores the need for personalized treatment strategies in ICU settings and highlights important areas for future research, including prospective studies to further elucidate these associations and their underlying mechanisms.

## Conclusions

This study has revealed pivotal insights into the role of age and comorbidity profiles in influencing the duration of mechanical ventilation among COVID-19 ICU patients, particularly highlighting the extended ventilation needs in older patients and those with specific comorbidities. These findings not only enrich our understanding of individual patient needs in critical COVID-19 care but also underscore the importance of personalized treatment approaches. The study's implications are profound, suggesting necessary revisions in clinical protocols for the management of critically ill COVID-19 patients and informing public health strategies for efficient resource allocation and pandemic preparedness. It underscores the urgent need for healthcare professionals and policymakers to integrate these nuanced insights into their ongoing response to the pandemic, advocating for adaptable and patient-centric treatment guidelines.
